# Supplementary material for: CNT-molecule-CNT (1D-0D-1D) van der Waals integration ferroelectric memory with 1-nm2 junction area
Source: Nat Commun. 2022 Aug 12;13:4556. doi: 10.1038/s41467-022-32173-8 (PMC9374722; doi:10.1038/s41467-022-32173-8)
Supplement: Supplementary file 1 — Supplementary Information [file 41467_2022_32173_MOESM1_ESM.pdf]

Supplementary Information

CNT-molecule-CNT (1D-0D-1D) van der Waals integration ferroelectric memory with 1-nm^2 junction area

Thanh Luan Phan[1,+], Sohyeon Seo[2,+], Yunhee Cho[2,3], Quoc An Vu[3,4], Young Hee Lee[3,4], Dinh Loc Duong[3,4,*], Hyoyoung Lee[2,3,*], and Woo Jong Yu[1,*]

Affiliations

[1]Department of Electrical and Computer Engineering, Sungkyunkwan University, Suwon 16419, Republic of Korea.

[2]Department of Chemistry, Sungkyunkwan University, Suwon 16419, Republic of Korea.

[3]Center for Integrated Nanostructure Physics (CINAP), Institute for Basic Science (IBS), Suwon 16419, Republic of Korea.

[4]Department of Energy Science, Sungkyunkwan University, Suwon 16419, Republic of Korea.

* Corresponding author. Email: D. L. Duong (ddloc@skku.edu), H. Y. Lee (hyoyoung@skku.edu), W. J. Yu (micco21@skku.edu)

[+] These authors contributed equally: Thanh Luan Phan, Sohyeon Seo

Contents

Table 1. Summary of device parameters and performance of representative molecule junctions

S1. Synthesis process of aryl azobenzene diazonium salts

S2. NMR spectra of diazonium salt and precursor

S3. FT-IR spectra of diazonium salt and precursor

S4. Molecular effect on the Raman spectra of CNT

S5. Electrical characteristics of CNT_B and CNT_B-M (with molecule layer)

S6. Molecular effect on the FTIR spectra of CNT and CNT with molecule layer

S7. Thickness change of CNT_B-M/CNT_T vdWI by trans-cis transition of azobenzene.

S8. Electrical characteristics of the pristine metallic-CNT

S9. Energy band diagrams of CNT_B-M/CNT_T device

S10. Energy barrier calculation

S11. Direct tunneling and Fowler-Nordheim tunneling calculation

S12. I-V characteristics of 4×4 crossbar array of CNT_B-M/CNT_T memory devices

S13. CNT_B-M/CNT_T vdWI fabrication process

Supplementary Table 1 Summary of device parameters and performance of representative molecule junctions.

Device structure	Active area (nm^2)	Current density (A/cm^2)	Yield (%)	On/off ratio	Memory function	Material dimensions	Reference (○)
**CNT-molecule-CNT**	**1**	**~3.4×10^8**	**100**	**~10^5**	**Yes**	**1D-0D-1D**	**This study**
Metal-molcule-metal	~10^6	2×10^2	95	#	No	3D-0D-3D	**Ref 56** Nature 441, 69-72 (2006)
Metal-molecule-metal	9×10^5	50	70-100	#	No	3D-0D-3D	**Ref 57** ACS Nano. 6, 9920-9931 (2012)
Metal-molecule-metal	2×10^{12}	30	#	#	No	3D-0D-3D	**Ref 4** Nat. Nanotechnol. 7, 438–442 (2012)
Metal-molecule-graphene	1.6×10^7	8×10^4	90	#	No	3D-0D-2D	**Ref 58** Adv. Mater. 23, 755–760 (2011)
Metal-molecule-metal nanoparticle	3600	5×10^3	95	#	No	3D-0D-0D	**Ref 44** Nature 559, 232-235 (2018)
Graphenen-molecule-graphene	9×10^{10}	0.01	80	10	Yes	2D-0D-2D	**Ref 43** Nat. Commun. 4, 1-7 (2013)

Graphene-molecule-graphene	25	3.6×10^5	#	10^2	No	2D-0D-2D	**Ref 5** Science, 352, 1443-1445 (2016)
Metal-molecule-metal	1×10^{10}	10^2	#	10	No	3D-0D-3D	**Ref 45** Adv. Mater. 20, 1467-1473 (2008)

The parameter is not specified in the literature; ○ Reference number in the main manuscript.

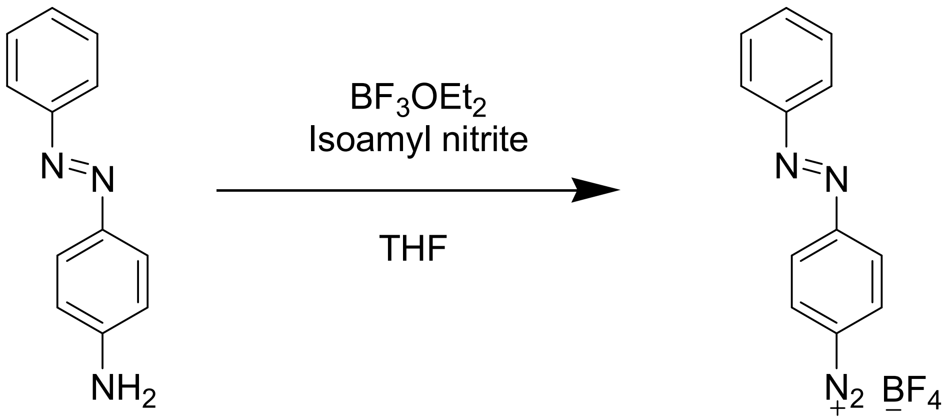

Supplementary Fig. 1 Synthesis process of azobenzene diazonium salts. Schematic of the synthesizing procedure for (E)-4-(phenyldiazenyl)benzene diazonium tetrafluoroborate.[1]

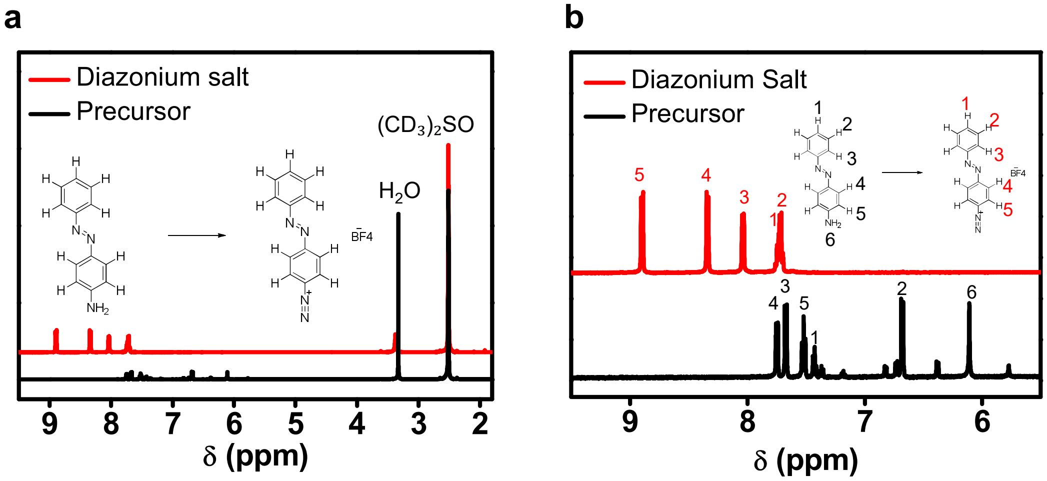

Supplementary Fig. 2 NMR spectra of azobenzene diazonium salts and precursor. a ^{1}H-NMR (500 MHz, ($C=O(CD_3)_2$) spectra of precursor and synthesized diazonium salt. **b** Expanded view for the range from 10 ppm to 5 ppm corresponding to x-axis in (a). The higher electronegativity of $-N_2^+$ compared to $–NH_2$ led to moving the ^{1}H NMR chemical shifts of azobenzene diazonium salt downward (increasing ppm) as shown in the ^{1}H-NMR spectra.

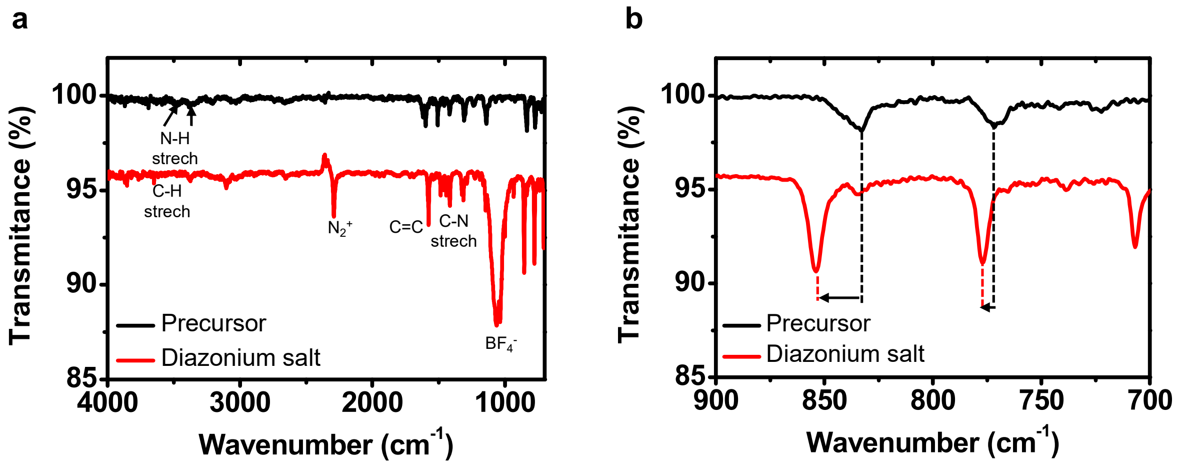

Supplementary Fig. 3 FT-IR spectra of diazonium salts and precursor. **a** FT-IR spectra of precursor and synthesized azobenzene diazonium salt. **b** Zoomed-in FT-IR spectra of (a) in the C-H aromatic range showing out of plane bending modes. Vibration frequencies are shifted to higher values owing to the higher electronegativity of the N_2^+ functional group.

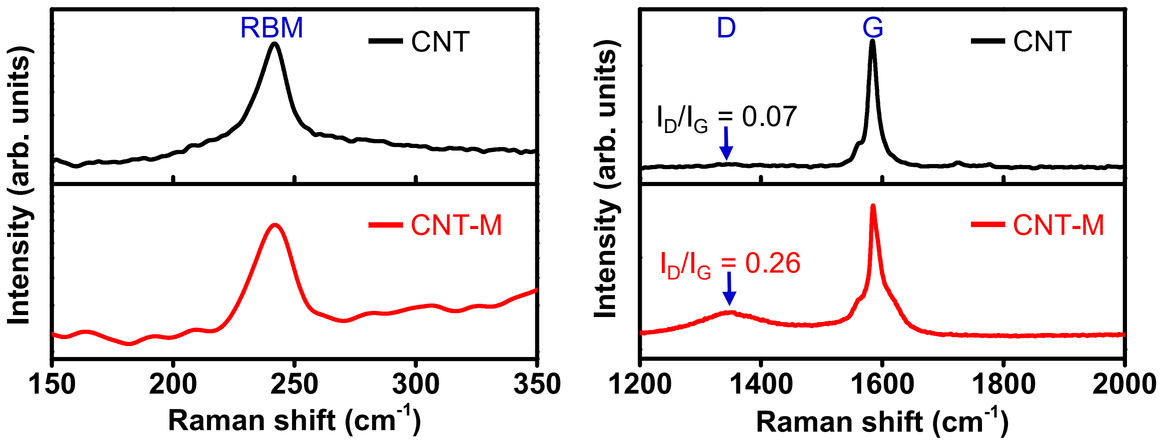

Supplementary Fig. 4 Molecular effect on the Raman spectra of CNT. Raman spectra of pristine CNT (black curve) and azobenzene molecule-modified CNT (CNT-M) (red curve).

Raman spectra of the pristine CNT (black curve) and CNT-M (red curve) were characterized at an excitation laser wavelength of 532 nm. The radial breathing mode (RBM) peak was narrowly centered at ~248 cm^{-1} (left panel), which originated from metallic SWCNT[2]. Regarding the inverse relationship ($\omega_{RBM} = 235.9/d_t + 5.5$) between the RBM peak frequency (ω_{RBM}) and the tube diameter (d_t),[3] the calculated diameters of the SWCNT are approximately 1 nm. The G-band (at 1590 cm^{-1}) (right panel) exhibits an obvious Lorentzian line shape in both CNT and CNT-M. However, a significant increase in the D peak intensity was exhibited for CNT-M (red) after the chemical reaction of the molecule and CNT, while a negligible D peak was exhibited in pristine CNT (black). This suggests that the molecules formed covalent C-C sp^3 bonds with CNT, leading to an increase of approximately 3.7 times in the I_D/I_G ratio (from 0.07 to 0.26).[4]

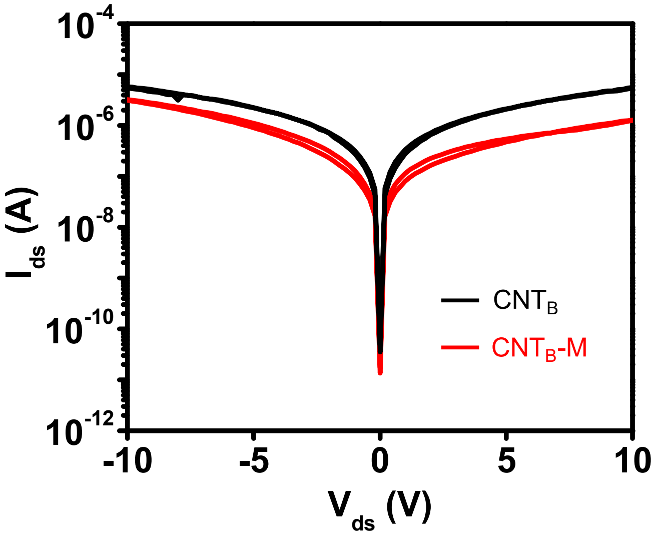

Supplementary Fig. 5 | Electrical characteristics of CNT_B and CNT_B-M (with molecule layer). The sweep directions of V_{ds} were from −10 V to 10 V (upward) and +10 V to −10 V (backward) for the original CNT_B (black curve) and CNT_B-M (red curve), respectively.

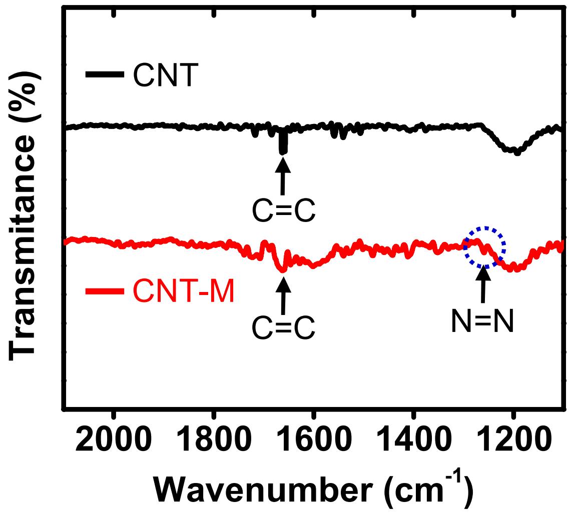

Supplementary Fig. 6 Molecular effect on the FTIR spectra of CNT and CNT-M (with molecule layer). FTIR spectra of pristine CNT (black curve) and azobenzene molecule-modified CNT (red curve). The C=C peak at ~1650 cm^{-1} belonging to SWCNT appears for both CNT (pristine) and CNT-M (with molecule), whereas the new peak of N=N for CNT-M was exhibited at 1236 cm^{-1} owing to the identical functional group of azobenzene after the reaction of SWCNT with azobenzene molecules.[4]

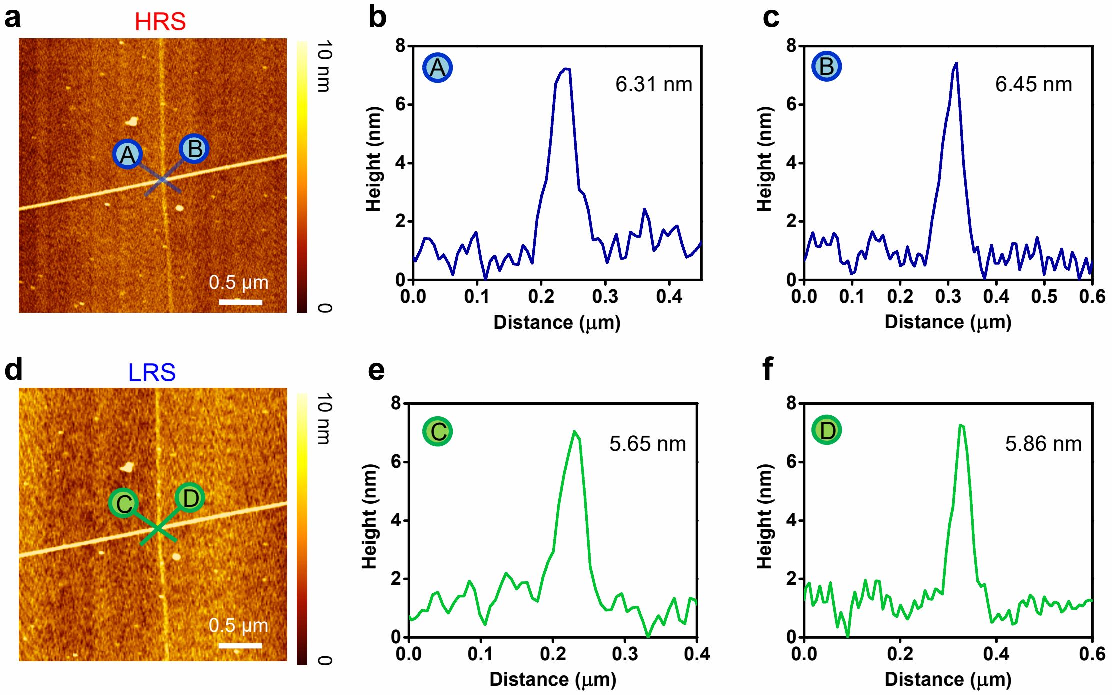

Supplementary Fig. 7 Thickness change of CNT_B-M/CNT_T vdWI by trans-cis transition of azobenzene. AFM image and height profile distribution of CNT_B-M/CNT_T vdWI at (**a-c**) HRS, and (**d-f**) LRS, respectively. The line profiles are displayed as cross-bar line as in (**a, d**).

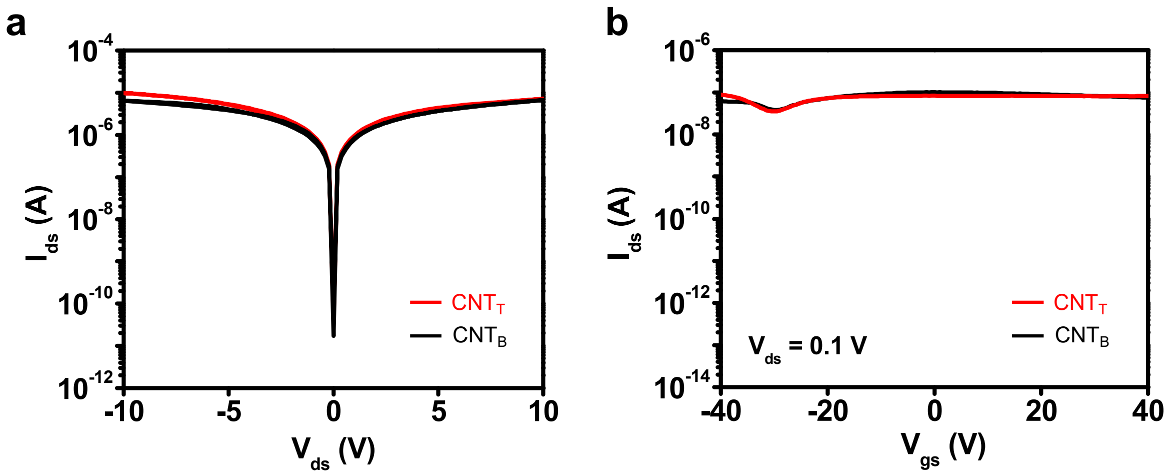

Supplementary Fig. 8 Electrical characteristics of the pristine metallic-CNT field effect transistor. a Output characteristic (I_{ds}-V_{ds}) curves of CNT_B (black) and CNT_T (red) over a range of V_{ds}= ±10 V. **b** Transfer curves (V_{gs}-I_{ds}) of CNT_B (black) and CNT_T (red) at V_{ds}=0.1 V and V_{gs}= ±40V. The metallic behavior of both prepared CNTs is confirmed, where no considerable current change was observed during a gate bias sweep.[5]

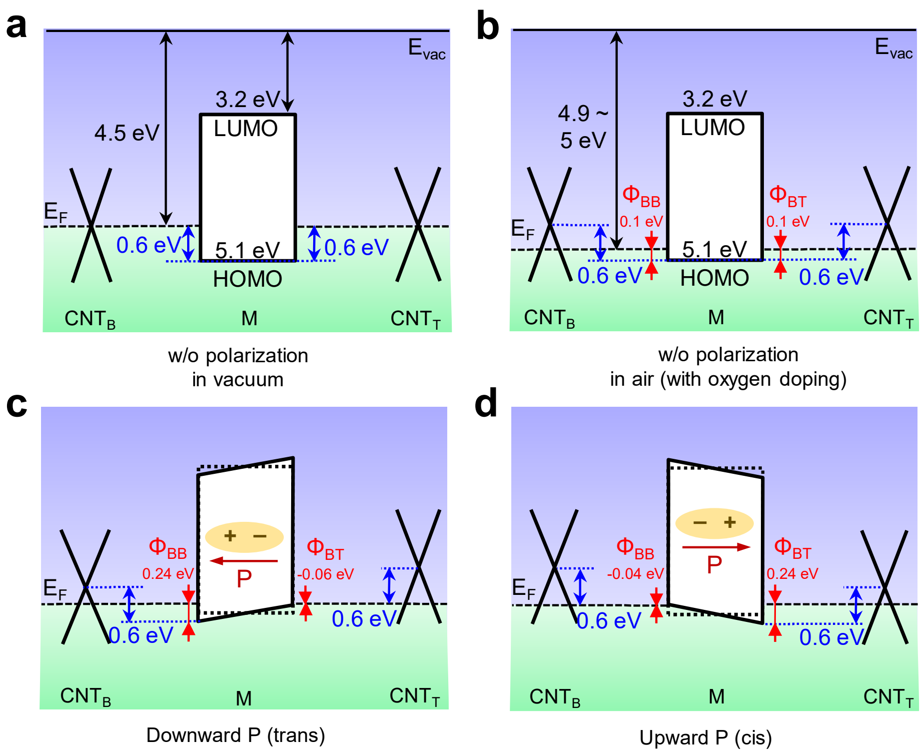

Supplementary Fig. 9 Energy band diagrams of CNT_B-M/CNT_T device. a-b Energy band diagrams of CNT_B-M/CNT_T device without polarization in (a) vacuum and (b) air. Φ_{BB} and Φ_{BT} are Schottky barrier height at CNT_B-molecule and CNT_T-molecule junctions, respectively. **c-d** Energy band diagram of CNT_B-M/CNT_T device under (c) downward (*trans*) and (d) upward (*cis*) polarization.

In Supplementary Fig. 9a, the highest occupied molecular orbital (HOMO: -5.1 eV) and the lowest unoccupied molecular orbital (LUMO: -3.2 eV) are obtained by DFT calculation, and the work function of m-CNT is theoretically about 4.5 eV in vacuum[6]. Majority carriers are hole carriers because the barrier height between m-CNT and LUMO (0.6 eV) is lower than m-CNT and HOMO (1.3 eV). Supplementary Fig. 9b shows the energy band diagram in air. It is known that the E_F of graphene[7,8] and m-CNT[9,10] can be shifted by doping or external field due to the finite density of states near the Dirac point, resulting in Schottky barrier height (Φ_B) change. In

our device, oxygen in the air donates hole carriers to the m-CNT[11], shifting E_F downwards (4.95~5.05 eV[12]) and reducing the barrier height.

Supplementary Fig. 9c and d show the barrier change under downward and upward ferroelectric polarization of azobenzene molecule, respectively. The ferroelectric dipole of azobenzene molecule shifts the E_F of CNT_B and CNT_T due to finite density of states near the Dirac point[9,10], resulting in Φ_B change. At the downward polarization (*trans* state, Supplementary Fig. 9c), electrons and holes are attracted to CNT_B and CNT_T by polarization field, resulting in upshifts and downshifts of E_F in CNT_B and CNT_T, respectively. Corresponding Schottky barrier height of CNT_B-Molecule (Φ_{BB}) and CNT_T–molecule (Φ_{BT}) increases and decreases by the E_F shift ($\Phi_B = 0.1$ eV + ΔE_F), respectively. The Φ_{BB} and Φ_{BT} are measured to 0.24 eV and -0.06 eV, respectively (Supplementary Fig. 10). At the upward polarization (*cis* state, Supplementary Fig. 9d), Φ_{BB} and Φ_{BT} are decreased to -0.04 eV and increased to 0.24 eV, respectively, by the same mechanism (Supplementary Fig. 10).

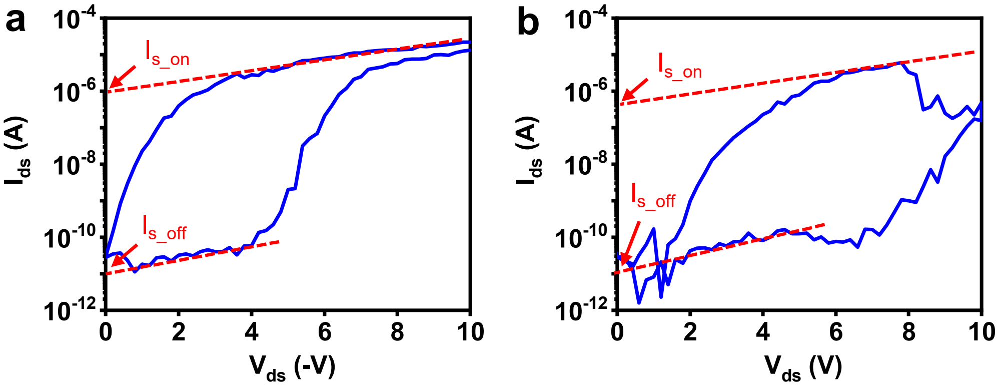

Supplementary Fig. 10 Energy barrier calculation. Saturation currents (I_s) for Schottky barrier calculation at (a) reverse and (b) forward region. Dashed lines are expansions of the linear region in the *I*–*V* curve, which intercept at $V_{ds} = 0$ V is saturation current density (I_s).

The thermionic current-voltage relationship of a Schottky barrier, neglecting series and shunt resistance, is given by (1)[13]

$$\mathrm{I} = AA^{*}T^{2}exp\left[\frac{-q\Phi_B}{kT}\right]\left(exp\left[\frac{qV}{nkT}\right]-1\right)=\mathrm{I}_S\left(exp\left[\frac{qV}{nkT}\right]-1\right) \quad (1)$$

Where I_s is the saturation current (obtained at Supplementary Fig. 10), A the diode area (1 nm^2), A^* Richardson's constant (120 A/cm^2K^2)[14], Φ_B the effective barrier height, q the electron charge, n the ideality factor, T the temperature, and V the applied drain voltage.

From $\mathrm{I}_S = AA^{*}T^{2}exp\left[\frac{-q\Phi_B}{kT}\right]$, the Schottky barrier height is calculated to (2)

$$\Phi_B = \frac{kT}{q}\ln\left(\frac{AA^{*}T^{2}}{I_S}\right) \quad (2)$$

At the reverse bias ($V_{ds} < 0$V), Φ_{BB} is 0.24 eV (i. downward P) and -0.04 eV (iv. upward P). At the forward bias ($V_{ds} > 0$V), Φ_{BT} is -0.06 eV (ii. downward P) and 0.24 eV (iii. upward P).

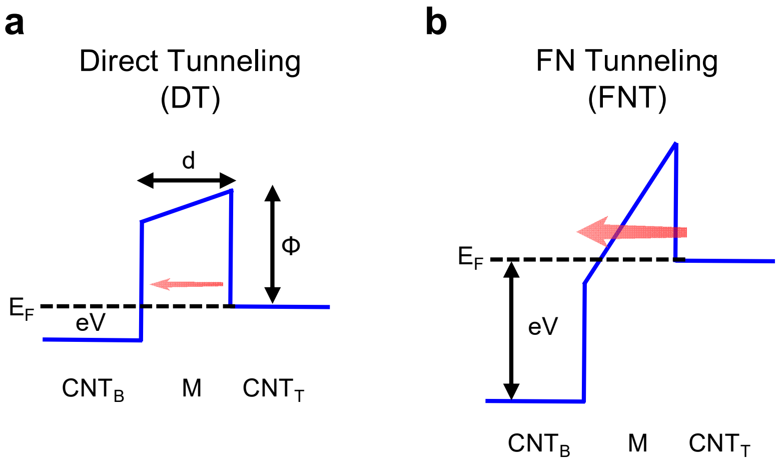

Supplementary Fig. 11 Direct tunneling (DT) and Fowler-Nordheim tunneling (FNT) calculation. a-b Schematic of the DT and FNT in the CNT_B-M/CNT_T device according to (a) low voltage regime and (b) high-voltage regime.

The relation between tunneling current (I) and the applied voltage (V_{ds}) can be expressed to (3)[15-17]

$$\mathrm{I} \propto \begin{cases} V_{ds} exp\left[\frac{-4\pi\sqrt{m^*\Phi_B}d}{h}\right] : \text{Direct tunneling} \\ {V_{ds}}^2 exp\left[\frac{-8\pi\sqrt{2m^*}\Phi_B^{\frac{3}{2}}d}{3hqV_{ds}}\right] : \text{FN tunneling} \end{cases} \quad (3)$$

By dividing V_{ds}^2 and making *ln()* in the equation, the relation between I and V_{ds} become (4)

$$\ln\left(\frac{\mathrm{I}}{V^2}\right) \propto \begin{cases} \ln\left(\frac{1}{V}\right) : \text{Direct tunneling} \\ -\left(\frac{1}{V}\right) : \text{FN tunneling} \end{cases} \quad (4)$$

Therefore, direct tunneling and FN tunneling show logarithmic and negative linear behavior in $ln(I/V^2)$ vs. $1/V$ plots, respectively (Fig. 3b and c).

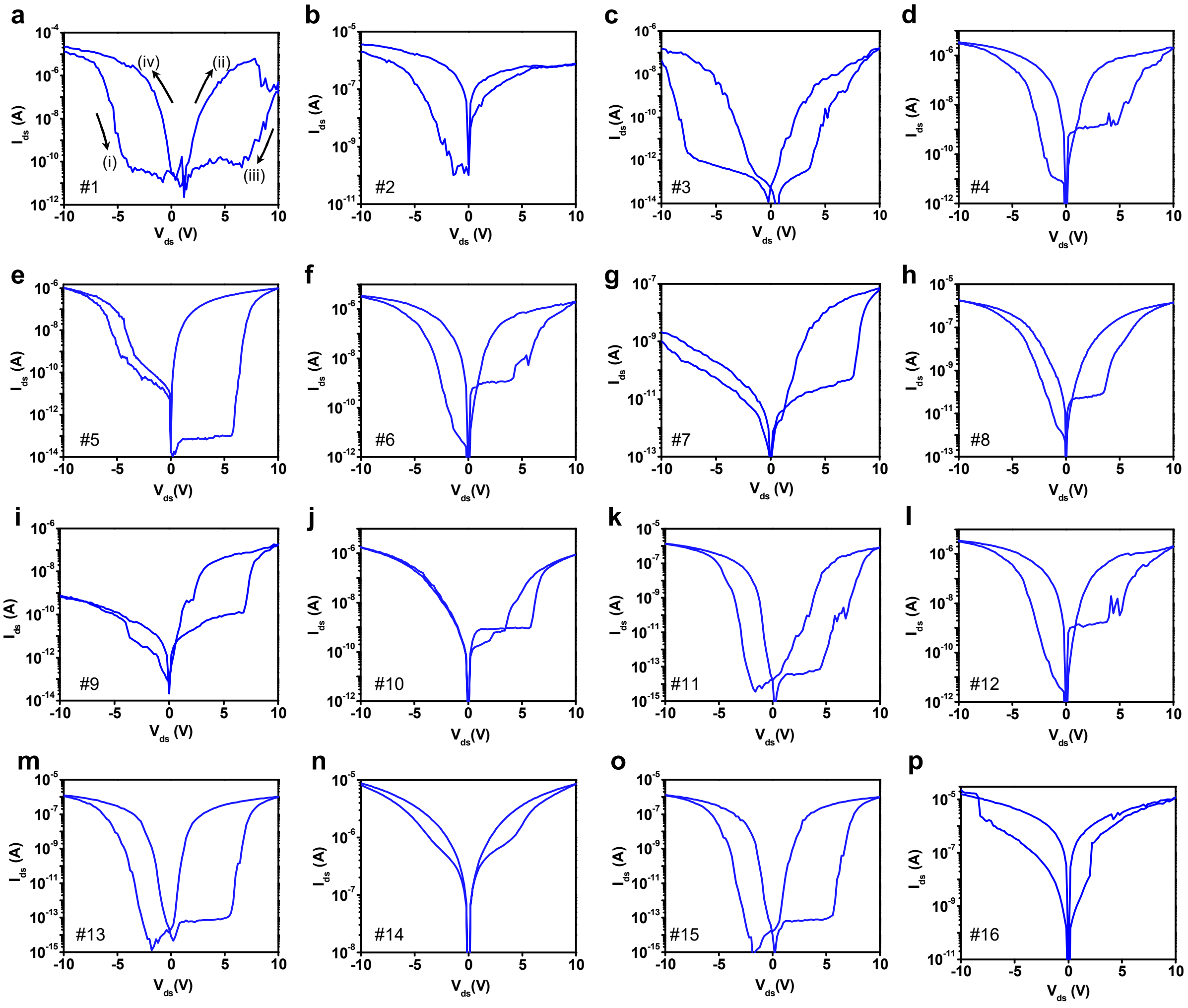

Supplementary Fig. 12 I-V characteristics of 4×4 crossbar array of CNT_B-M/CNT_T memory devices. a-p Electrical response of CNT_B-M/CNT_T vdWI according to 16 active domain devices numbered from #1 to #16, respectively, corresponding to Fig. 4d (right panel) for forward (i, ii), and reverse (iii, iv) scans.

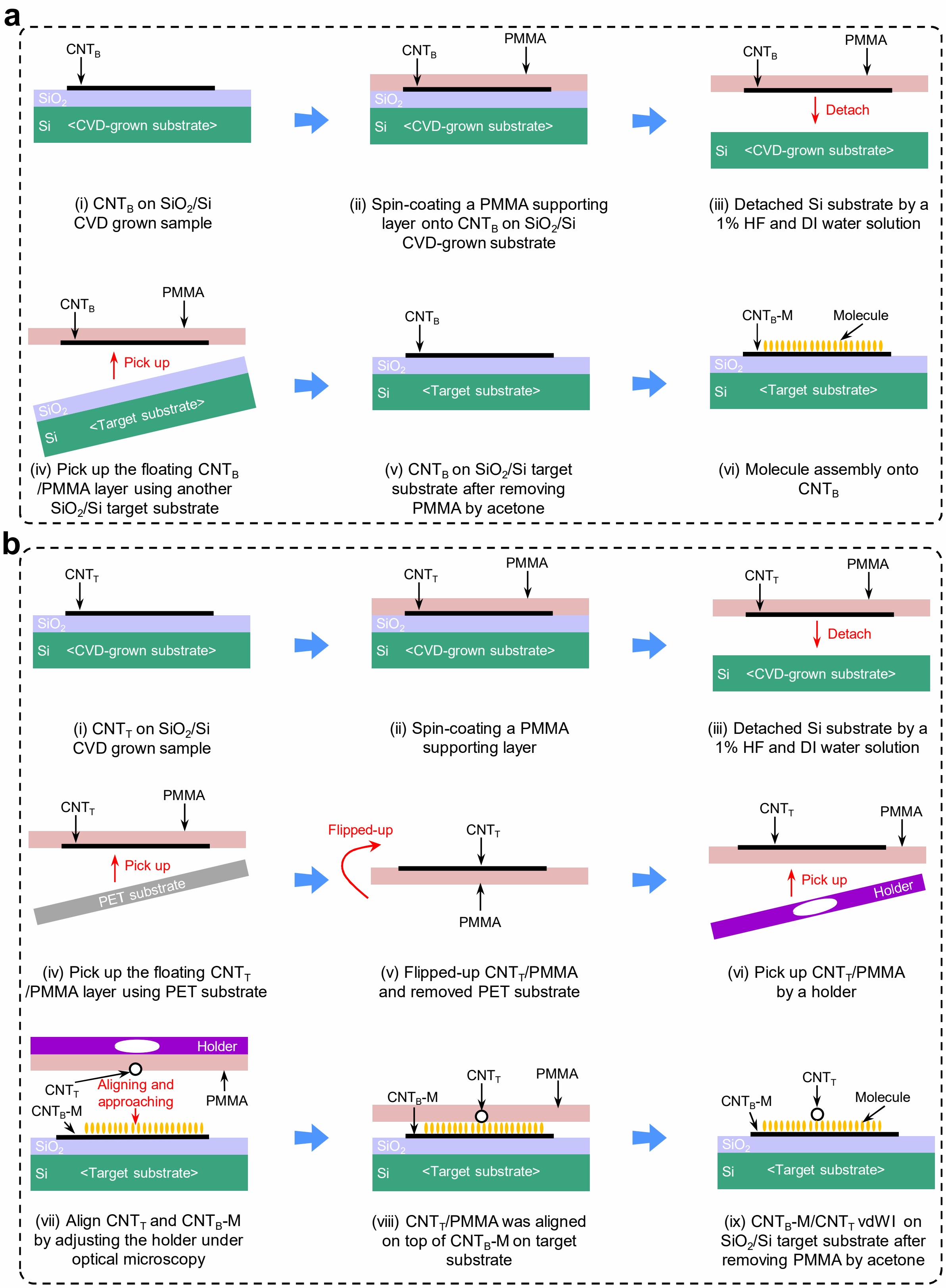
a
CNT$_B$
SiO_2
Si <CVD-grown substrate>
(i) CNT$_B$ on SiO_2/Si CVD grown sample
PMMA
(ii) Spin-coating a PMMA supporting layer onto CNT$_B$ on SiO_2/Si CVD-grown substrate
Detach
(iii) Detached Si substrate by a 1% HF and DI water solution
Pick up
Si <Target substrate>
(iv) Pick up the floating CNT$_B$ /PMMA layer using another SiO_2/Si target substrate
(v) CNT$_B$ on SiO_2/Si target substrate after removing PMMA by acetone
CNT$_B$-M
Molecule
(vi) Molecule assembly onto CNT$_B$
b
CNT$_T$
(i) CNT$_T$ on SiO_2/Si CVD grown sample
(ii) Spin-coating a PMMA supporting layer
(iii) Detached Si substrate by a 1% HF and DI water solution
PET substrate
(iv) Pick up the floating CNT$_T$ /PMMA layer using PET substrate
Flipped-up
(v) Flipped-up CNT$_T$/PMMA and removed PET substrate
Holder
(vi) Pick up CNT$_T$/PMMA by a holder
Aligning and approaching
(vii) Align CNT$_T$ and CNT$_B$-M by adjusting the holder under optical microscopy
(viii) CNT$_T$/PMMA was aligned on top of CNT$_B$-M on target substrate
(ix) CNT$_B$-M/CNT$_T$ vdWI on SiO_2/Si target substrate after removing PMMA by acetone

Supplementary Fig. 13 CNT_B-M/CNT_T vdWI fabrication process. **a** Wet-transfer technique used for CNT_B. **b** Dry transfer technique to align CNT_T on CNT_B-M to form the CNT_B-M/CNT_T vdWI device.

Supplementary References

1. Min, M., Bang, G. S., Lee, H. & Yu, B. C. A photoswitchable methylene-spaced fluorinated aryl azobenzene monolayer grafted on silicon. *Chem. Commun.* **46**, 5232–5234 (2010).

2. Zhang, D. et al. (n,m) Assignments and quantification for single-walled carbon nanotubes on SiO_2/Si substrates by resonant Raman spectroscopy. *Nanoscale* **7**, 10719–10727 (2015).

3. Jorio, A. et al. Structural (n , m) Determination of Isolated Single-Wall Carbon Nanotubes by Resonant Raman Scattering. *Phys. Rev. Lett.* **86**, 1–4 (2001).

4. Seo, S., Min, M., Lee, S. M. & Lee, H. Photo-switchable molecular monolayer anchored between highly transparent and flexible graphene electrodes. *Nat. Commun.* **4**, 1–7 (2013).

5. Phan, T. L. et al. Efficient Gate Modulation in Screening-Engineered MoS_2/Single-Walled Carbon Nanotube Network Heterojunction Vertical Field-Effect Transistor. *ACS Appl. Mater. Interfaces* **11**, 25516–25523 (2019).

6. Barone, V. Peralta, J. E., Uddin, J. & Scuseriaa, G. E. Screened exchange hybrid density-functional study of the work function of pristine and doped single-walled carbon nanotubes. *J. Chem. Phys.* **124**, 024709 (2006).

7. Yu, W. J. *et al.* Vertically stacked multi-heterostructures of layered materials for logic transistors and complementary inverters. *Nat. Mater.* **12**, 246–252 (2013).

8. Yu, W. J. *et al.* Highly efficient gate-tunable photocurrent generation in vertical

heterostructures of layered materials. *Nat. Nanotechnol.* **8**, 952–958 (2013).

9. Li, X. *et al.* Gate-tunable contact-induced Fermi-level shift in semimetal. *Proc. Natl. Acad. Sci.* **119**, e2119016119 (2022).

10. Zhang, J. *et al.* Carbon-nanotube-confined vertical heterostructures with asymmetric contacts. *Adv. Mater.* **29**, 1–8 (2017).

11. Kang, D. Park, N. Ko, J. Bae, E. & Park, W. Carbon-nanotube-confined vertical heterostructures with asymmetric contacts. *Nanotechnology* **16**, 1048–1052 (2005).

12. Shiraishi, M. Ata, M. Work function of carbon nanotubes. *Carbon* **39** 1913–1917 (2001).

13. Schroder, D. K. Semiconductor material and device characterization. *Hoboken, New Jersey, USA: John Wiley & Sons, Inc.* (2006).

14. Wei, X. Wang, S. Chen, Q. & Peng, L. Breakdown of Richardson's law in electron emission from individual self-joule-heated carbon nanotubes. *Sci. Rep.* 4:5102, DOI: 10.1038/srep05102 (2014).

15. Simmons, J. G. Generalized formula for the electric tunnel effect between similar electrodes separated by a thin insulating film. *J. Appl. Phys.* 34, 1793 (1963).

16. Vu, Q. A. *et al.* Tuning carrier tunneling in van der Waals heterostructures for ultrahigh detectivity. *Nano Lett.* **17**, 453–459 (2017).

17. Ikuno, T. *et al.* Electron transport properties of Si nanosheets: Transition from direct tunneling to Fowler-Nordheim tunneling. *Appl. Phys. Lett.* **99**, 023107 (2011).
